# Single machine scheduling problems with sequence-dependent setup times and precedence delays

**DOI:** 10.1038/s41598-022-13278-y

**Published:** 2022-06-08

**Authors:** Shih-Wei Lin, Kuo-Ching Ying

**Affiliations:** 1 Department of Information Management, Chang Gung University, Taoyuan, 333 Taiwan, ROC; 2 Department of Emergency Medicine, Keelung Chang Gung Memorial Hospital, Keelung City, 204 Taiwan, ROC; 3 Department of Industrial Engineering and Management, Ming Chi University of Technology, New Taipei City, 243 Taiwan, ROC; 4 Department of Industrial Engineering and Management, National Taipei University of Technology, Taipei, 106 Taiwan, ROC

**Keywords:** Engineering, Mathematics and computing

## Abstract

Sequence-dependent setup times and precedence delays occur frequently in various production environments. This study investigates the single machine scheduling problem with setup times and precedence delays that occur in an amplifier assembly company. This study proposes a novel mixed-integer linear programming model and a lean iterated greedy algorithm to minimize the makespan for this problem. Based on the property of delayed precedence constraints, the lean iterated greedy (LIG) algorithm uses a simple but effective lean construction mechanism that can discard infeasible solutions to reduce the waste of unnecessary searches and quickly converge to the (near) global optimum. The computational results show that LIG significantly outperforms the state-of-the-art algorithm in terms of solution quality and computational efficiency. This study mainly contributes to providing a simple, effective, and efficient algorithm that can facilitate industrial applications and serve as a new benchmark approach for future research.

## Introduction

Scheduling orders for a single machine, a production line, an entire manufacturing system, or a manufacturing system with a bottleneck station are an example of the single machine scheduling problem (SMSP)^1^. In recent decades, SMSPs have attracted much attention. This study aimed to optimize the makespan of the SMSP with sequence-dependent setup times (SDSTs) and delayed precedence (DP), which is a significant (sub) problem in production scheduling^2^. The SDST constraint occurs when the setup times of jobs depend on their immediate predecessors. Explicitly considering the SDST constraint in production planning decisions can lead to significant savings in setup costs, the elimination of waste, and increased productivity in many real industrial settings, especially in the textile, printing, chemical, pharmaceutical, and metallurgical industries^3,4^.

The DP constraint, also called a precedence delay or a non-negative time-lag, means that certain pairs of jobs require a delay between the completion time of the predecessor and the start time of the successor. This constraint usually occurs when the workpiece must be processed multiple times on the machine due to precedence constraints, waiting for its temperature to drop, or its adhesive/paint to dry^2^. Using the three-field notation scheme proposed by Graham^5^, the problem can be signified as $1|s_{ij} ,{\text{prec}}(d_{ij} )|C_{\max }$, where 1 means that the shop type is a single machine; $s_{ij}$ and ${\text{prec}}(d_{ij} )$ represent the scheduling problem with the SDST and DP constraints, respectively; and $C_{\max }$ denotes the scheduling objective is to minimize the makespan, which is directly related to machine utilization and used in most scheduling problems.

Compared to other constraints, there is little research on SMSPs with DP constraints. The literature review has shown that Wikum et al.^6^ was the first to explicitly address SMSPs with DP constraints. They considered the minimization of various objective functions in SMSPs subject to minimum DP constraints, maximum DP constraints, or a combination of the two DP constraints for different types of precedence relations. Wikum et al.^6^ proved that except for the simplest of precedence relations, most of these SMSPs are *NP*-hard, and then proposed a number of results, including polynomial algorithms for some special cases, heuristic methods, and the worst-case bounds of two SMSPs with minimum DP constraints. However, their study did not consider SDST constraints.

Balas et al.^7^ examined an SMSP with release dates, delivery times, and DP constraints without SDSTs constraints. They first pointed out that this problem is a relaxation of the job-shop scheduling problem (JSP) that is tighter than the standard SMSP relaxation. Then, Balas et al.^7^ proposed a modified shifting bottleneck procedure that uses relaxation to minimize makespan in JSPs. The computational results showed that this approach could consistently provide better solutions for all classes of JSPs compared to other algorithms, but with more computational time. Finta and Liu^8^ investigated an SMSP with PD constraints, with the goal of minimizing the makespan. In a scheduling problem with DP constraints, the release dates (RDs) of jobs are unknown before scheduling. They applied the concept of DP constraints to model the RDs of the jobs and computed them as a function of a given feasible schedule. Finta and Liu^8^ proved that the problem is *NP*-hard in the strong sense when the delay and execution times are integers, it is polynomially solvable with an *O*(*n*^2^) optimal algorithm. However, SDST constraints were not considered in this study either. Du and Han^9^ presented an improved heuristic for solving SMSPs with DP constraints. Their research showed that this new approach has a better worst-case performance ratio of 4/3 than the heuristic of Wikum et al.^6^, which provided the best worst-case guarantee among all heuristics searching for insertion schedules. Moreover, the heuristic can optimally solve the corresponding unit execution time problem. Schuurman^10^ proposed a pseudo-polynomial algorithm and a fully polynomial approximation scheme to solve an SMSP with PD constraints that has a simple tree-type structure, with the objective of minimizing the makespan. Schuurman proved that the proposed fully polynomial approximation scheme can provide a good approximation to the optimal schedule for the considered problem.

Brucker et al.^11^ studied an SMSP with start-start DP constraints that allow positive and negative time-lags between the start times of two jobs. Unfortunately, SDSTs were not considered in their study. They pointed out that some complex job-shop and open-shop scheduling problems involving multiprocessor tasks, general purpose machines, or changeover times can be reduced to such an SMSP. Brucker et al.^11^ showed that SMSPs with start-start relations are a complex problem and developed a branch and bound (B&B) algorithm to solve SMSPs with arbitrary time-lags and the reduction problems of the classical job-shop and open-shop scheduling problems. However, the computational results showed that the transformation and the B&B algorithm cannot efficiently solve the classical job-shop and open-shop scheduling problems. Munier and Sourd^12^ studied three sub-problems of SMSPs with DP constraints in which each operation has at most one predecessor and one successor. They first presented an algorithm to solve the makespan minimization problem when all operations have the same duration and all delays are equal to a constant. Then, they considered that the length of each delay is smaller than the shortest processing time and presented one algorithm for minimizing the makespan and another for minimizing the flow time when all processing times are equal.

Muthusamy et al.^13^ considered an SMSP with fuzzy time delays and fuzzy precedence constraints in which schedules were evaluated on three different performance measures: the makespan, the degree of satisfaction with the time delays, and the degree of satisfaction with the fuzzy precedence. They showed that the problem of finding a set of non-dominated feasible schedules that contains exactly one schedule from each equivalence class of non-dominated feasible schedules can be solved in polynomial time. Brucker et al.^14^ studied SMSPs with constant processing times and generalized precedence constraints in the form of chains with constant delays. They showed that the problem of minimizing the total completion time is polynomially solvable. They also gave a compact encoding of an optimal schedule for minimizing the makespan of the problem. Given that a general SMSP with PD constraints is NP-hard, Xie et al.^15^ studied an SMSP with fuzzy PD constraints and fuzzy processing times, with the objective of minimizing the makespan. They employed the upper and lower bounds presented by Wikum et al.^6^ as parameters of L-R type fuzzy numbers and showed that the problem is polynomially solvable using the modified Lawler’s algorithm in *O*(*n*^2^). Yuan and Ou^16^ proposed an *O*(*n*^2^) algorithm for solving bi-criteria SMSPs with fuzzy time delays and fuzzy precedence constraints, in which the schedules were evaluated by performance measures of the degree of satisfaction with the fuzzy time delays and the degrees of satisfaction with the fuzzy PD constraints. However, it was left to the decision maker to decide which of the non-dominated schedules is better. Recently, Zhang et al.^17^ proposed heuristics and branch-and-bound algorithms to minimize the makespan for SMSPs with PD constraints, release times, and delivery times. Their computational results showed that the proposed approaches could achieve significant improvements in running time and number of iterations on test instances both with and without delayed precedence constraints.

Inspired by a practical problem in a company that assembles amplifiers, Kuo et al.^2^ presented the first study of the $1|s_{ij} ,{\text{prec}}(d_{ij} )|C_{\max }$ problem. The process of assembling amplifiers consists of several steps, each of which is performed sequentially. Since different tools, fixtures, and assembly components are needed for different parts or products, SDSTs are required for preprocessing all parts or products. Since the covers of amplifiers are assembled with a special adhesive, there are DP constraints on assembly because of the need to wait for the adhesive to dry. Kuo et al.^2^ presented a nonlinear programming model, a variable neighborhood search (VNS) metaheuristic, and five simulated annealing (SA) algorithms to solve the $1|s_{ij} ,{\text{prec}}(d_{ij} )|C_{\max }$ problem. The computational results, based on 16 randomly generated scenarios, showed that VNS outperformed the five SA algorithms in solving the $1|s_{ij} ,{\text{prec}}(d_{ij} )|C_{\max }$ problem and provided robust solutions for different DP scenarios.

The above review of the relevant literature showed there is a dearth of research on SMSPs that considers both SDSTs and DP constraints. To bridge the gap between scheduling theory and practical applications, this study proposed a mixed-integer linear programming (MILP) model and a more effective and efficient lean iterated greedy (LIG) algorithm to solve the $1|s_{ij} ,prec(d_{ij} )|C_{\max }$ problem. Since VNS is the best available metaheuristic, it was used as a benchmarking algorithm in this study. The rest of this paper is organized as follows. In “Problem description and MILP model formulation” section , the $1|s_{ij} ,prec(d_{ij} )|C_{\max }$ problem was defined, and its MILP model was formulated. Section "The lean iterated greed algorithm" describes the proposed LIG algorithm in detail. The experimental results and statistical analysis based on different scenarios are presented in "Computational experimentation and results" section. Finally, concluding remarks and recommendations for future research are given in Section "Concluding remarks".

## Problem description and MILP model formulation

The $1|s_{ij} ,{\text{prec}}(d_{ij} )|C_{\max }$ problem consists of a finite set *J* of *n* independent jobs to be processed on a single machine. The processing time of job $j$ $(j = 1, \, ..., \, n)$ on the machine is $p_{j}$. An SDST,$s_{ij}$, is needed before processing job *j*, if it is processed immediately after job *i*. *A* is the set of DP constraints for all jobs. For certain job pairs, ${(}i,j{)} \in {\mathbf{\rm A}}$, and the succeeding job *j* cannot be processed until its predecessor has completed the job and the corresponding delay time, $d_{ij}$, between the completion time of the preceding job *i* and the start time of the job has elapsed. The objective is to find a feasible schedule for these *n* jobs that satisfies the precedence delays to minimize the makespan,$C_{\max }$, i.e., the completion time of the last executed job. In addition, the following assumptions are made in the addressed $1|s_{ij} ,prec(d_{ij} )|C_{\max }$ problem:

- Processing times, SDSTs, and delay times are assumed to be non-negative integers.
- Each job is assumed to be available for processing immediately at the beginning of the planning period.
- Each job must be processed on the machine exactly once without preemption.
- Each job is independent of the others and its processing time is known, fixed, and finite.
- Each job is processed as early as possible. Thus, there are no intentional waiting times for jobs or idle times of the machine.
- The machine is available at the beginning of the planning period.
- The machine can process at most one job at a time.
- The machine is continuously available to process jobs throughout the planning period, and there are no interruptions due to breakdowns, maintenance, or any such cause.
- The machine has an adequate waiting area where jobs can wait before being processed.

In this study, a novel MILP model was developed to formulate the $1|s_{ij} ,prec(d_{ij} )|C_{\max }$ problem. The following notations were used in the proposed formulation:

### Indices

$j$,$i$ Job tags, $j = 1,2, \, ..., \, n{; }i = 0,1, \, ..., \, n$, where 0 is a dummy job

*A* Set of delayed precedence constraints of all jobs

### Parameters

$n$ Number of jobs

$b_{j}$ Starting time of job $j$

$s_{ij}$ Sequence-dependent setup times required for the processing of job $j$ after job $i$

$d_{ij}$ Delay time needed for the processing of job $j$ after job $i$

$p_{j}$ Processing time of job $j$

*M* A sufficiently large positive number

### Decision variables

$$x_{ij}$$ Binary variable, $$x_{ij} = \left\{ \begin{gathered} 1,{\text{ if job }}j{\text{ is processed immediately after job }}i{; } \hfill \\ {\text{0, otherwise}}{.} \hfill \\ \end{gathered} \right.$$

$c_{i}$,$c_{j}$ Completion time of job *i* and job *j*

With the above preliminaries and notations, the MILP model of the $1|s_{ij} ,prec(d_{ij} )|C_{\max }$ problem could be formulated as follows:

1 $${\text{Minimize}}\;\;C_{\max }$$

subject to

2 $$C_{\max } \ge c_{j} ; \, j = 1,2, \, ..., \, n,$$

3 $$\sum\limits_{j = 0}^{n} {x_{ij} = 1;} \, i = 0,1, \, ..., \, n{ (}i \ne j),$$

4 $$\sum\limits_{i = 0}^{n} {x_{ij} = 1;} \, j = 1,2, \, ..., \, n{ (}i \ne j),$$

5 $$b_{j} \ge c_{i} + s_{ij} - M(1 - x_{ij} ); \, i = 1,2, \, ..., \, n; \, j = 1,2, \, ..., \, n{ (}i \ne j),$$

6 $$b_{j} \ge c_{i} + d_{ij} ; \, \forall (i,j) \in A,$$

7 $$c_{j} = b_{j} + p_{j} ; \, j = 1,2, \, ..., \, n,$$

8 $$x_{ij} \in \{ 0,1\} ; \, i = 0,1, \, ..., \, n{; }j = 1,2, \, ..., \, n{ (}i \ne j).$$

Constraint (1) specifies the objective function of the problem. Constraint set (2) calculates the makespan. Constraint sets (3) and (4) ensure that each job is dispatched to only one position in the sequence. Constraint sets (5) and (6) describe the relationship between the start time of a job and the completion time of its predecessor. Constraint set (7) describes the relationship between the completion time of a job and its start time. Finally, constraint set (8) defines the ranges of the decision variable $x_{ij}$.

## The lean iterated greed algorithm

In recent decades, researchers have proposed various meta-heuristics for solving intractable combinatorial optimization problems. Among these meta-heuristics, the iterative greedy (IG) algorithm^18^ is one of the most efficient and effective methods to be successfully used in solving various scheduling problems^19–23^. The original IG algorithm proposed earlier^18^ consists of two key phases: destruction and reconstruction. In each iteration, some incumbent solution elements are first selected and removed in the destruction phase. In the subsequent reconstruction phase, all removed elements are sequentially inserted into the remaining partial solution using a greedy heuristic until a new solution is assembled. After a new solution is constructed, some acceptance criteria are used to evaluate whether the incumbent solution will be replaced by it or not. The above process is repeated until certain termination conditions are met.

Based on the basic framework of IG and the property of DP constraints, this study presented an LIG algorithm to solve the $1|s_{ij} ,prec(d_{ij} )|C_{\max }$ problem. The innovation of the LIG algorithm is the proposed lean construction mechanism. To reduce the waste of unnecessary searches, two properties are used in the lean construction mechanism to discard infeasible positions that violate the DP constraints in the reconstruction phase of LIG, so that the search process can stay within the feasible solution space and quickly converge to the (near) global optimum. The following subsections elaborate on the solution representation, the construction of the DP constraint table, the method for computing the makespan, the lean construction mechanism, and the detailed procedures of the LIG algorithm.

### Solution representation

 In the proposed LIG algorithm, a feasible solution, $\Pi$, is encoded as a string of *n* numbers representing the job permutation without violating the DP constraints. For example, a solution encoded as [1 7 2 3 5 8 6 4] means that it has eight jobs, and the job permutation is 1-7-2-3-5-8-6-4. The following subsections explain how to ensure the generated solution does not violate the DP constraints.

### Construction of the DP constraints table

In this study, a DP constraint table was created to quickly determine whether or not inserting a removed job into a specific position in the partial solution would violate the DP constraint. Suppose that each DP constraint is expressed as $DPC(i,j) = d_{ij}$, which means that job *j* cannot be processed before job *i* and its start time should be delayed at least $d_{ij}$ time units after the completion time of job *i*. Then, the DP constraint table with *n* jobs can be constructed as follows:

- ***Step 1*** As shown in Table 1, the grid (*i*, *j*) is filled with *U* (undetermined) as the initial value if the predecessor, job *i*, is not equal to the successor, job *j*; otherwise, it is filled with *P* (prohibited) as the initial value.
- ***Step* 2** For each pair of jobs with $DPC(i,j) = d_{ij}$, the grid (*i, j*) and the corresponding grid (*j, i*) are refilled with $d_{ij}$ and *P* (prohibited), respectively. If any job *k* must be processed after job *j*, the grid (*k, i*) is refilled with *P*.

**Table 1:** Initial values fill in the DP constraints table.

Predecessor\successor	Job 1	Job 2	$\cdots$	Job *n *− 1	Job *n*
Job 1	*P*	*U*	*U*	*U*	*U*
Job 2	*U*	*P*	*U*	*U*	*U*
$\vdots$	$\vdots$	$\vdots$	$\vdots$	$\vdots$	$\vdots$
Job *n *− 1	*U*	*U*	*U*	*P*	*U*
Job *n*	*U*	*U*	*U*	*U*	*P*

For example, suppose there are eight jobs with five DP constraints, as shown in Table 2. Since the first DP constraint is $DPC(1,2) = 35,$ the grid (1, 2) and the corresponding grid (2, 1) are refilled with 35 and *P*, respectively. The second DP constraint is $DPC(2,3) = 0$, and grids (2, 3) and (3, 2) are refilled as 0 and *P*, respectively. Since job 3 also must be processed after job 1, the grid (3, 1) is refilled with *P.* In the same way, processing the three remaining DP constraints yields the final lag times of the DP constraints table, as shown in Table 3.

**Table 2:** DP constraints of the example.

No.	Constraint
1	${\text{DPC}}(1,2) = 35$
2	${\text{DPC}}(2,3) = 0$
3	${\text{DPC}}(3,4) = 13$
4	${\text{DPC}}(5,6) = 24$
5	${\text{DPC}}(7,8) = 46$

**Table 3:** Final lag times of the DP constraints table.

Predecessor\successor	Job 1	Job 2	Job 3	Job 4	Job 5	Job 6	Job 7	Job 8
Job 1	*P*	35	*U*	*U*	*U*	*U*	*U*	*U*
Job 2	*P*	*P*	0	*U*	*U*	*U*	*U*	*U*
Job 3	*P*	*P*	*P*	13	*U*	*U*	*U*	*U*
Job 4	*P*	*P*	*P*	*P*	*U*	*U*	*U*	*U*
Job 5	*U*	*U*	*U*	*U*	*P*	24	*U*	*U*
Job 6	*U*	*U*	*U*	*U*	*P*	*U*	*U*	*U*
Job 7	*U*	*U*	*U*	*U*	*U*	*U*	*P*	46
Job 8	*U*	*U*	*U*	*U*	*U*	*U*	*P*	*P*

### The makespan calculation method

**Figure 1:**
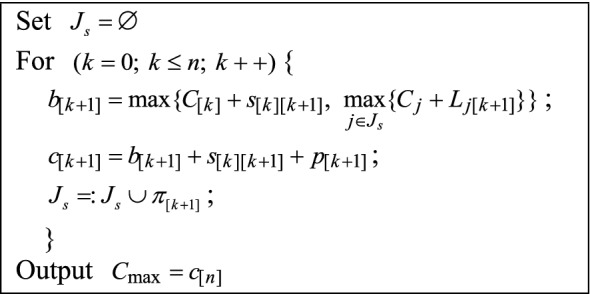
The procedure of the makespan calculation.

 Assume that $J_{s}$ is the set of scheduled jobs, $L_{j[k + 1]}$ means the lag time of the grid (*j*, *k* + 1) in the DP constraints table, and $\pi_{[j]}$ ($j = 0, \, 1 \, , \, 2, \, ..., \, n$) denotes the job scheduled at the *j*^th^ position of a given feasible solution, $\Pi = (\pi_{[0]} , \, \pi_{[1]} , \, ..., \, \pi_{[n]} )$, where $\pi_{[0]}$ is a dummy job with $p_{[0]} = 0$ and $s_{[0][1]} = 0$. Then, the makespan of the given feasible solution $\Pi$ can be calculated using the procedure shown in Fig. 1.

Using the DP constraints table and SDST data respectively shown in Tables 3 and 4 as an example, the makespan of a given feasible solution, $\Pi = (1, \, 7, \, 2, \, 3, \, 5, \, 8, \, 6, \, 4)$, can be calculated as shown in Table 5.

**Table 4:** The sequence-dependent setup times of the example.

Predecessor\successor	Job 1	Job 2	Job 3	Job 4	Job 5	Job 6	Job 7	Job 8
Job 1	–	8	8	9	5	5	4	6
Job 2	6	–	9	9	7	8	6	4
Job 3	9	5	–	7	7	6	4	6
Job 4	8	8	7	–	6	6	5	4
Job 5	4	5	9	5	–	9	8	3
Job 6	6	6	6	7	6	–	4	8
Job 7	5	5	6	4	4	3	–	3
Job 8	7	5	6	5	6	4	4	–

**Table 5:** Calculation of the makespan for given feasible solution.

Job	SDST	Lag time	Start time	Processing tine	Completion time
1	0	0	0	12	12
7	4	0	16	12	28
2	5	14	47	11	58
3	9	0	67	14	81
5	7	0	88	16	104
8	3	0	107	15	122
6	4	2	128	14	142
4	7	0	149	17	166

**The lean construction mechanism.** To avoid creating infeasible solutions that violate any DP constraints and to converge quickly to the (near) optimal solution, the proposed LIG algorithm implements a simple but effective lean construction mechanism described below.

Assuming there are *k* jobs in the current partial solution, there are *k* + 1 possible positions to insert the job that was removed in the previous destruction phase. Some positions may violate the DP constraints. Therefore, it is not necessary to test all positions and select the best position to insert the removed job. The following two properties are implemented in the lean construction mechanism to reduce the waste of unnecessary searches. First, if we insert the removed job at a position immediately before job $J_{b}$ in the partial solution will violate the DP constraints; it also violates the DP constrants if we insert the removed job at any position before $J_{b}$. On the other hand, if we insert the removed job at a position immediately after job $J_{a}$ in the partial solution will violate the DP constraints; it also violates the DP constraints if we insert the removed job at any position after $J_{a}$. Using the lean construction mechanism, infeasible positions that violate the DP constraints can be discarded in the reconstruction phase of LIG, and the solution quickly can converge to the (near) optimum.

**Figure 2:**
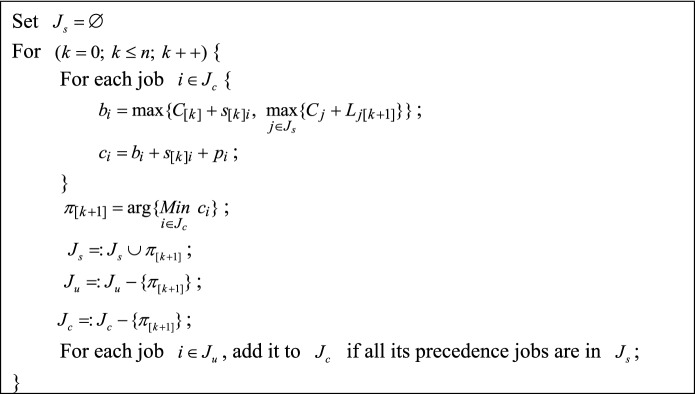
The detailed procedure of constructing the initial solution.
Step 2 Enter the destruction and reconstruction phases
  - Destruction phase: Randomly select $D$ jobs from the incumbent solution $\Pi_{incumbent}$, then move them to $\Pi_{d}$ and sort them in the order in which they were selected. Set the remaining jobs in the incumbent solution as the current partial solution, $\Pi_{p}$.
  - Reconstruction phase: Apply the lean construction mechanism to sequentially insert the jobs from $\Pi_{d}$ into $\Pi_{p}$ until a new complete solution,$\Pi_{new}$, is created.
Step 3 Apply the acceptance criteria
  Apply the following criteria to evaluate whether or not the incumbent solution $\Pi_{incumbent}$ and the best-found solution $\Pi_{best}$ will be updated by $\Pi_{new}$:
  IF $C_{\max } (\Pi_{new} ) \le C_{\max } (\Pi_{best} )$, set $\Pi_{best} : = \Pi_{new}$ and $\Pi_{incumbent} : = \Pi_{new}$;
  ELSE_IF $$C_{\max } (\Pi_{new} ) \le C_{\max } (\Pi_{incumbent} )$$, set $$\Pi_{incumbent} : = \Pi_{new}$$;
  ELSE_IF $$C_{\max } (\Pi_{new} ) > C_{\max } (\Pi_{incumbent} )$$, generate *r* ~ U (0,1);
  IF $$r < Exp( - \Delta E)$$, set $$\Pi_{incumbent} : = \Pi_{new}$$.
  Otherwise, discard $$\Pi_{new}$$.
  Here, $C_{\max } ( \cdot )$ denotes the makespan of a specific solution $( \cdot )$; $r \in [0,1]$ is a random number generated from the uniform distribution U(0,1); $\Delta E = [C_{\max } (\Pi_{new} ) - C_{\max } (\Pi_{incumbent} )]/[SF \times C_{\max } (\Pi_{incumbent} )]$, wherein $SF$ is a scale factor used to control the probability of accepting a worse solution.
Step 4 Apply the stopping criterion
  Repeat Steps 2 to 3 until the maximum allowable computational time, $T_{\max }$, is reached. In this study, $T_{\max } = \tau \times n$ (CPU time in seconds), in which $\tau$ is a parameter that controls the maximum allowable computational time.
  Using the data presented in Tables 2, 3, 4 as an example, one iteration of the proposed LIG algorithm is shown in Fig. 3 to clearly illustrate the procedure. In Step 1, a feasible initial solution, $\Pi =$(1, 7, 2, 3, 5, 8, 6, 4) with $C_{\max } (\Pi ) = 166$, was generated. In Step 2, the destruction and reconstruction phases, which can be considered as the perturbation mechanism, were executed. In Step 2(a), three jobs (*i.e*., jobs 8, 5, and 1) were randomly selected and sequentially removed from the incumbent solution to $\Pi_{d}$. In Step 2(b), a new complete solution, $\Pi_{new} =$(1, 7, 5, 2, 3, 6, 4, 8) with $C_{\max } (\Pi_{new} ) =$ 150, was created by sequentially inserting the jobs in $\Pi_{d} =$(8, 5, 1) into $\Pi_{p} =$(7, 2, 3, 6, 4). Then, in Step 3, $\Pi_{best}$ and $\Pi_{incumbent}$ were updated by $\Pi_{new}$ according to the acceptance criteria. If the quality of $\Pi_{new}$ was worse than that of $\Pi_{incumbent}$, the Boltzmann function ($e^{{[C_{\max } (\Pi_{new} ) - C_{\max } (\Pi_{incumbent} )]/[SF \times C_{\max } (\Pi_{incumbent} )]}}$) was used to determine whether $\Pi_{incumbent}$ could be replaced by $\Pi_{new}$ or not. This mechanism is usually implemented in the annealing process of the SA algorithm to make it easier for the incumbent solution to escape from the local optimum.

**Procedures of the proposed LIG algorithm.** The procedures of the proposed LIG algorithm are described as follows:

Step 1 Generate a feasible initial solution
  The method for generating the initial solution is to add one job at a time to the last position of the current partial solution until a complete schedule is created. To ensure the generated initial solution is feasible, the job with the shortest completion time that satisfies the DP constraints is selected next in each iteration. The detailed procedure for generating the initial solution is shown in Fig. 2, where $J_{c}$ is the set of candidate jobs that satisfies the DP constraints in each iteration and $J_{u}$ denotes the set of unscheduled jobs.

**Figure 3:**
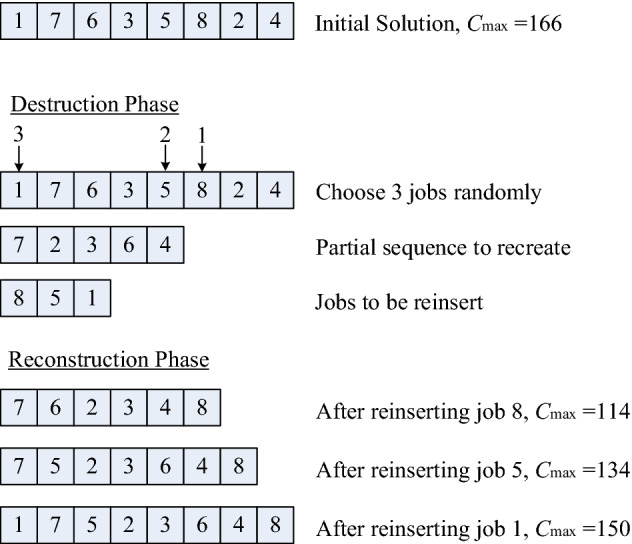
An example for one iteration of the LIG algorithm.

## Computational experimentation and results

An extensive computational experiment was conducted to evaluate the performance of the proposed IG algorithm in solving the $1|s_{ij} ,prec(d_{ij} )|C_{\max }$ problem. The following subsections describe the benchmark problem set, parameter calibration, and computational results of LIG compared to the state-of-the-art algorithm.

### The benchmark problem set

 To fairly compare the performance of the proposed LIG algorithm with that of the existing best solution algorithm, *i.e.*, VNS, the benchmark problem set of a similar approach by Kuo et al.^2^ was created and used in this study. The benchmark problem set consisted of 540 test instances created as described here. The number of jobs was $n =${10, 15, 20, 25, 50, 75, 100}; the processing times were randomly generated using the uniform distributions [10, 20] and [20, 30], respectively; the SDSTs were randomly generated using the uniform distributions [5, 10] and [10, 15], respectively; the delay times were randomly generated using the uniform distributions [20, 40] and [40, 60], respectively; and $\left\lceil {n/2} \right\rceil$ number of randomly selected jobs were subject to precedence constraints, in which $\left\lceil {n/4} \right\rceil$ number of jobs had zero delay time. On this basis, ten test instances were generated for each of the $7 \times 2 \times 2 \times 2 \times 1 \times 1 = 56$ configurations to analyze the performance of LIG under different operational scales and workloads. The files of these test instances can be downloaded from http://swlin.cgu.edu.tw/data/SDSTsDP_Data.7z.

The proposed LIG algorithm and VNS were coded and compiled in Visual C +  + (2017), and the source code of VNS was provided by Kuo et al.^2^. All experiments were performed on a personal computer (PC) with the following specifications: an Intel Core Xeon CPU E5-1620v2 @ 3.70 GHz processor, 64 GB RAM, and the Windows 10 operating system. The MILP mathematical model was solved using Gurobi version 9.0 on the same PC with a maximum computation time of 3600 s for each test instance. The final solution generated by the Gurobi MILP solver was recorded as a feasible solution.

**Parameter calibration.** Before starting the computational experiments, 12 test instances were generated using the same data generation procedures as described above to calibrate the three parameters, *D*, *SF*, and $\tau ,$ of the LIG algorithm. For this purpose, as shown in Table 6, four alternatives for each parameter were tested to analyze which results were better. Each of the 12 test instances was solved 20 times in the preliminary tests. The resulting maximum (Max.), average (Ave.), and minimum (Min.) makespan values for each parameter combination were recorded, and their respective relative percent deviations (*RPDs*) were calculated as follows:

$$RPD = (C_{\max } (\Pi )^{\min } - C_{\max } (\Pi )^{P} )/C_{\max } (\Pi )^{\min } \times 100\%$$

where $C_{\max } (\Pi )^{P}$ is the makespan value yielded by the parameter combination *P,* and $C_{\max } (\Pi )^{\min }$ denotes the minimum makespan value yielded among all parameter combinations.

**Table 6:** Parameter values in the calibration experiments.

Parameter	Value tested
*SF*	10, 100, 1000, 10,000
*D*	2, 3, 4, 5
$\tau$	0.05, 0.10, 0.15, 0.20

**Figure 4:**
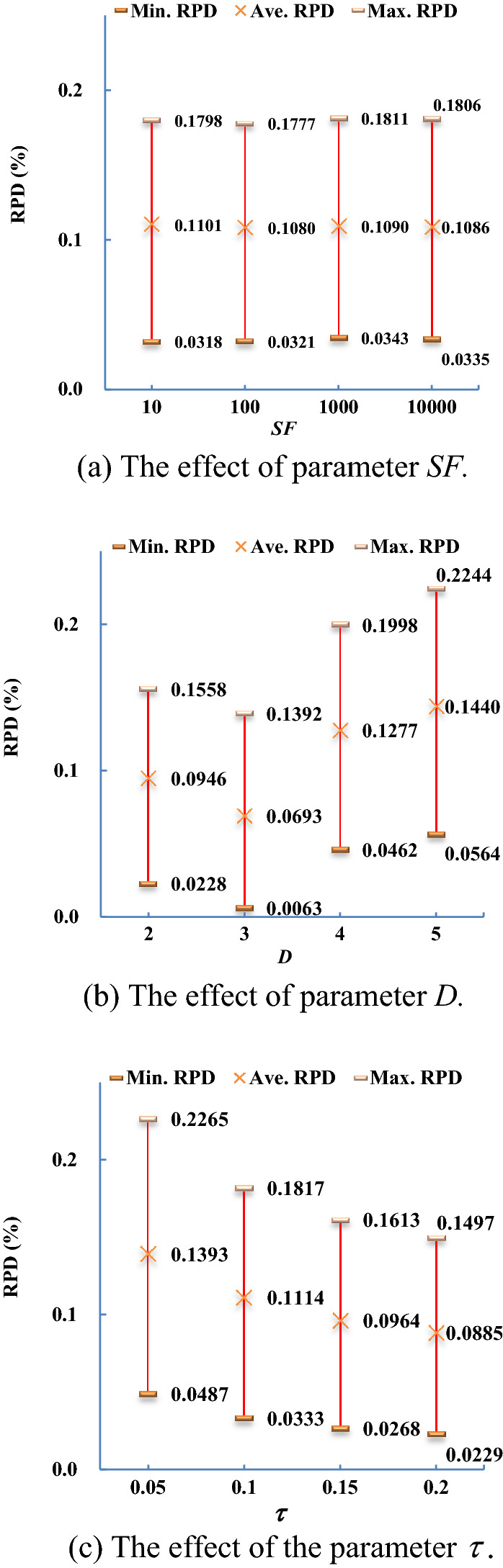
The effect of each parameter on the solution quality.

Figure 4 shows the effects of each parameter on the quality of the solutions. The parameter *SF* affects the probability of accepting a worse solution. In general, the higher the value of *SF*, the higher the probability of accepting a worse solution, and the slower the convergence speed of the LIG algorithm. However, if the value of *SF* is too small, the probability that a worse new solution will be accepted will also be very low, and the algorithm will not be able to escape from the local optimum. As can be seen in Fig. 4a, if the value of *SF* is too small, the corresponding Ave. *RPD* will be slightly larger. However, there is no significant difference between the different *SF* values. The value of *D* indicates the number of jobs removed in each iteration of the destruction phase, which may affect the range of the neighborhood solution search. Figure 4b demonstrates that the average *RPD* is larger when the value of *D* is too high or too low. Thus, when the value of *D* is too small, the search range of the neighborhood solution is too small, resulting in the poor quality of the solution. However, if too many jobs are removed, a better solution may not be obtained because the maximum computation time is limited. Figure 4c shows that the higher the value of $\tau$, the smaller the value of Ave. *RPD*. This means that a better solution can be obtained with more computation time. Based on the computation time and the quality of the solutions in the preliminary experiments,$SF = 100,$ $D = 3,$ and $\tau = 0.1$ were selected for the final experiments.

## Results and discussion

In this study, the average relative percentage deviation (Ave. *RPD*) was used as a performance measure to compare the performance of the LIG and VNS algorithms. For each small instance ($n =$ 10 and 15), the *RPDs* of the solutions obtained using LIG, VNS, and the MILP mathematical model were calculated according to the following expression:

$${\text{RPD}} = \frac{{C_{{{\text{max}}}}^{{{\text{Method}}}} - C_{{{\text{max}}}}^{{{\text{LB}}}} }}{{C_{{{\text{max}}}}^{{{\text{LB}}}} }} \times 100\%$$

where $C_{{{\text{max}}}}^{{{\text{Method}}}}$ is the best makespan value of the feasible solution obtained with a given method among five trials, and $C_{\max }^{LB}$ is the lower bound (LB) of the makespan value obtained by the MILP mathematical model with a maximum computation time of 3600 s for each test instance. A feasible solution was optimal when the gap between its makespan value and the LB was zero.

Table 7 summarizes the computational results for the small instances and shows that the total average *RPD* value of the solutions obtained with LIG was smaller than those obtained with the VNS and the MILP model. The MILP model obtained optimal solutions in 80 out of 80 and one out of 80 test instances for the test instances using $n =$ 10 and $n =$ 15, respectively. It is noteworthy that all optimal solutions obtained with the MILP model were also obtained with LIG. Overall, LIG obtained eight and 93 better solutions than the MILP model and VNS, respectively, for 160 small instances. The CPU times required by LIG and VNS were significantly shorter than those of the MILP model. It could be concluded that the tested metaheuristics were better for solving small instances when the efficiency of the approach was the most important indicator. These analytical results confirmed that the proposed LIG algorithm had excellent convergence to optimal solutions compared to the VNS algorithm and the MILP model.

**Table 7:** Average *RPD*s (%) and running time for small-scale instances.

*n*	Processing Time	SDST	Delay Time	MILP		LIG		VNS	
				RPD	Time	RPD	Time	RPD	Time
10	[10, 20]	[5, 10]	[20,40]	0.000	3.76	0.000	1.002	0.597	4.56
10	[10, 20]	[5, 10]	[40,60]	0.000	2.96	0.000	1.010	0.052	4.45
10	[10, 20]	[10, 15]	[20,40]	0.000	5.51	0.000	1.009	0.238	4.44
10	[10, 20]	[10, 15]	[40,60]	0.000	3.12	0.000	1.009	0.574	4.53
10	[10, 20]	[5, 10]	[20,40]	0.000	4.83	0.000	1.009	0.196	4.47
10	[20, 30]	[5, 10]	[40,60]	0.000	3.73	0.000	1.009	0.663	4.42
10	[20, 30]	[10, 15]	[20,40]	0.000	6.91	0.000	1.009	0.000	4.48
10	[20, 30]	[10, 15]	[40,60]	0.000	4.35	0.000	1.007	0.433	4.49
15	[20, 30]	[5,10]	[20,40]	1.181	3600.02	1.079	1.509	2.801	5.44
15	[10, 20]	[5, 10]	[40,60]	1.141	3359.48	1.105	1.509	3.416	5.47
15	[10, 20]	[10, 15]	[20,40]	0.782	3600.02	0.756	1.510	1.723	5.49
15	[10, 20]	[10, 15]	[40,60]	0.950	3600.02	0.894	1.510	2.288	5.44
15	[20, 30]	[5, 10]	[20,40]	0.618	3600.03	0.618	1.509	1.412	5.42
15	[20, 30]	[5, 10]	[40,60]	0.776	3600.02	0.731	1.510	1.984	5.45
15	[20, 30]	[10, 15]	[20,40]	0.531	3600.02	0.512	1.509	1.256	5.41
15	[20, 30]	[10, 15]	[40,60]	0.611	3600.02	0.611	1.508	1.573	5.48
Total Ave				0.412	1787.18	0.394	1.259	1.200	4.96

The numerical results were also elaborated for solving larger test instances. The $1|s_{ij} ,prec(d_{ij} )|C_{\max }$ problem is very complex, so high-quality feasible solutions and LBs could not be obtained with the MILP model for the test instances with $n =$ 20, 25, 50, 75, and 100. Therefore, the *RPD* value for each larger test instance was calculated using the following expression:

$${\text{RPD}} = \frac{{C_{{{\text{max}}}}^{{{\text{Method}}}} - C_{{{\text{max}}}}^{{{\text{best}}}} }}{{C_{{{\text{max}}}}^{{{\text{best}}}} }} \times 100\%$$

where $C_{{{\text{max}}}}^{{{\text{Method}}}}$ is the best makespan value of the solution obtained with a given method among five trials, and $C_{\max }^{\text{best}}$ is the best makespan value among the solutions obtained with LIG and VNS.

Table 8 lists the computational results for the larger test instances and shows that the best and mean *RPD*s of the solutions obtained with LIG were significantly smaller than those obtained with VNS with respect to all categories of the larger test instances. The total average best and mean *RPD*s obtained with the proposed LIG algorithm were 0.000% and 0.050%, respectively, while those obtained with VNS were 3.279 and 3.580%, respectively. The mean *RPD*s achieved with LIG were smaller than those of VNS, indicating that the performance of LIG was more robust than that of VNS at all larger production scales and workloads. In addition, the computation time required by LIG was much less than that of VNS. It was observed that the gap between the computation times of LIG and VNS increased as the number of jobs increased.

**Table 8:** Average *RPD*s (%) and running time for larger test instances.

Category	LIG			VNS		
	Best *RPD*s	Mean *RPD*s	Time (s)	Best *RPD*s	Mean *RPD*s	Time (s)
***n***						
25	0.000	0.059	2.50	2.891	3.229	8.43
50	0.000	0.066	4.98	4.842	5.194	19.54
75	0.000	0.045	7.48	5.218	5.490	34.16
100	0.000	0.045	9.98	0.620	0.806	40.01
**Processing time**						
[10, 20]	0.000	0.056	5.39	3.841	4.224	22.54
[20, 30]	0.000	0.037	5.41	2.567	2.803	21.25
SDSTs						
[5, 10]	0.000	0.053	5.39	3.533	3.866	22.00
[10, 15]	0.000	0.040	5.41	2.874	3.159	21.79
**Delay times**						
[20, 40]	0.000	0.045	5.39	3.140	3.439	21.85
[40, 60]	0.000	0.048	5.41	3.265	3.585	21.94
Total Ave.	0.000	0.050	5.73	3.279	3.580	23.35

As a final step in the numerical analysis, one-sided paired *t*-tests were performed on the best and mean *RPD*s to validate whether or not LIG was significantly superior to VNS. The statistical results are summarized in Table 9. From Table 9, it could be seen that the statistical tests at a confidence level of $\alpha =$ 0.05 demonstrated the proposed LIG algorithm was significantly superior to the VNS algorithm in terms of best and mean *RPD*s for both small and large test instances.

**Table 9:** Statistical results from paired-*t* tests ($\alpha =$ 0.05).

Problem Size	Type	IG *vs*	VNS
Small	Best RPD	Paired difference (*RPD*)	$-$ 0.1717
		*t*-value	$-$ 7.6120
		Degree of freedom	159
		*P*-value	0.0000
Small	Mean RPD	Paired difference (*RPD*)	$-$ 0.6172
		*t*-value	$-$ 18.5008
		Degree of freedom	159
		*P*-value	0.0000
Larger	Best RPD	Paired difference (*RPD*)	$-$ 0.6774
		*t*-value	$-$ 49.9303
		Degree of freedom	399
		*P*-value	0.0000
Larger	Mean RPD	Paired difference (*RPD*)	$-$ 0.9443
		*t*-value	$-$ 59.2770
		Degree of freedom	399
		*P*-value	0.0000

## Concluding remarks

The $1|s_{ij} ,{\text{prec}}(d_{ij} )|C_{\max }$ problem is a theoretical and practical problem that corresponds to conditions in many existing production systems. This study contributed to the literature by proposing a novel MILP model and an LIG algorithm that filled a gap in the development of solution methods for this understudied problem. Numerical experiments have shown that LIG consistently found better solutions than the benchmarking algorithm in all test instances, at a lower computational cost, making it a viable solution approach for relevant industrial applications.

Despite its importance for real-world applications, research on the $1|s_{ij} ,{\text{prec}}(d_{ij} )|C_{\max }$ problem is still scarce and can be extended in several directions. First, more effective and efficient exact methods and heuristic algorithms, such as those based on supervised- and unsupervised-learning-based algorithms, should be further explored. Second, an alternative performance measure or multi-criteria SMSP with SDSTs and DP constraints are worthy of research. Third, from a practical point of view, new extensions of the $1|s_{ij} ,{\text{prec}}(d_{ij} )|C_{\max }$ problem, such as multi-agent problems, deserve further exploration. Finally, the extension of this deterministic problem to stochastic and dynamic models is also worth further investigating.

## Data Availability

The datasets generated and analyzed during the current study are available at http://swlin.cgu.edu.tw/data/SDSTsDP_Data.7z.
